# Assessment of a primary care-based telemonitoring intervention for home care patients with heart failure and chronic lung disease. The TELBIL study

**DOI:** 10.1186/1472-6963-11-56

**Published:** 2011-03-08

**Authors:** Iñaki Martín-Lesende, Estibalitz Orruño, Carmen Cairo, Amaia Bilbao, José Asua, María I Romo, Itziar Vergara, Juan C Bayón, Roberto Abad, Eva Reviriego, Jesús Larrañaga

**Affiliations:** 1 Bilbao Primary Health Care Region, Osakidetza - Basque Health Service, Bizkaia, Spain; 2 Basque Office for Health Technology Assessment (OSTEBA), Department of Health and Consumer Affairs, Basque Government, Vitoria-Gasteiz, Spain; 3 Basque Foundation for Health Innovation and Research (BIOEF), Bizkaia, Spain; 4 Knowledge Management and Evaluation, Department of Health and Consumer Affairs, Basque Government, Vitoria-Gasteiz, Spain

## Abstract

**Background:**

Telemonitoring technology offers one of the most promising alternatives for the provision of health care services at the patient's home. The primary aim of this study is to evaluate the impact of a primary care-based telemonitoring intervention on the frequency of hospital admissions.

**Methods/design:**

A primary care-based randomised controlled trial will be carried out to assess the impact of a telemonitoring intervention aimed at home care patients with heart failure (HF) and/or chronic lung disease (CLD). The results will be compared with those obtained with standard health care practice. The duration of the study will be of one year. Sixty patients will be recruited for the study. In-home patients, diagnosed with HF and/or CLD, aged 14 or above and with two or more hospital admissions in the previous year will be eligible.

For the intervention group, telemonitoring will consist of daily patient self-measurements of respiratory-rate, heart-rate, blood pressure, oxygen saturation, weight and body temperature. Additionally, the patients will complete a qualitative symptom questionnaire daily using the telemonitoring system. Routine telephone contacts will be conducted every fortnight and additional telephone contacts will be carried out if the data received at the primary care centre are out of the established limits. The control group will receive usual care. The primary outcome measure is the number of hospital admissions due to any cause that occurred in a period of 12 months post-randomisation. The secondary outcome measures are: duration of hospital stay, hospital admissions due to HF or CLD, mortality rate, use of health care resources, quality of life, cost-effectiveness, compliance and patient and health care professional satisfaction with the new technology.

**Discussion:**

The results of this study will shed some light on the effects of telemonitoring for the follow-up and management of chronic patients from a primary care setting. The study may contribute to enhance the understanding of alternative modes of health care provision for medically unstable elderly patients, who bear a high degree of physical and functional deterioration.

**Trial Registration:**

ISRCTN: ISRCTN89041993

## Background

It is estimated that by 2020, chronic diseases will contribute to more than 60% of illnesses requiring treatment [1]. Frequently, several chronic diseases occur at the same time in the same patients, often elderly individuals. This patient group, progressively growing in size, is characterised by a high use of health care resources, elevated rates of hospitalisations and a high prevalence of disability and dependence during the course of the disease [1-4]. Elderly chronic patients frequently experience multiple hospital admissions and such exacerbations have a major impact on the quality of life. There is good evidence that early identification of the exacerbations that arise from heart failure (HF) and chronic lung disease (CLD) reduces the risk for hospital admission and improves the quality of life of these chronic patients [5,6].

In the view of the ageing population and the growing impact of chronic diseases, there is a pressing need to adapt and to search for new approaches to health care. In this sense, the used of information and communication technology (ICT) applied to the monitoring of chronic patients provides new health care alternatives. Thus, novel methods for the delivery of quality health care could increase the effectiveness of chronic disease management while containing costs and using scarce human resources to maximum effect. Home telemonitoring allows the transmission of patients' vital signs once or more per day and provides diagnostic information to health care professionals. Telemetric-supported self-monitoring of HF and CLD has the potential to promote self-care, improve compliance, enable timely response to deterioration and improve the follow-up after hospital discharge [7,8]. A number of studies have shown that telemonitoring improves the quality of life of patients and their families [9,10]. From an economic view point, home telemonitoring of chronic patients appears to reduce health care costs [11-14]. Nevertheless, higher-quality economic studies are required to give greater insights into the potential cost-effectiveness of home telemonitoring [15].

Home telemonitoring has been found to reduce rates of hospitalisation and emergency department visits for chronic obstructive pulmonary disease (COPD) patients, while findings for hospital bed days were not consistent and varied between studies [15,16]. There was a higher mortality-rate among patients with COPD using home telemonitoring compared with usual care, but the number of original studies was scarce and the sample sizes were relatively small. Hence, the outcomes must be interpreted cautiously [15,16]. According to recent systematic reviews, telemonitoring interventions for patients with HF have been shown to be effective in reducing the risk of all-cause mortality, HF-related hospitalisations and emergency department visits [15,17-19]. Moreover, the use of home telemonitoring for patients with HF improved quality of life, patient self-care and evidence-based prescribing [15,17-19]. However, further studies of high methodological quality are required to give more precise information about the potential clinical benefits of home telehealth interventions prior to scaling-up the use of the new technology [7,19].

The telemonitoring system used in the present study is based on a telemonitoring system employed in another hospital-based clinical trial [20]. The home telemonitoring system has been successfully tested on patients with HF and COPD in a randomised controlled trial carried out in Donostia University Hospital (Basque Country), *Current Controlled Trials *ISRCTN62033748. In contrast to the aforementioned trial, in the present study the management of the telemonitoring intervention is carried out by primary care nurses and general practitioners (GPs). To the best of our knowledge, no other primary care-based tele-homecare studies have been undertaken with regards to the type of patients and the telemonitoring procedure employed in this investigation.

### Hypothesis and objectives of the study

The hypothesis that we set out to test in this study is that telemonitoring controlled by a primary care team can benefit home care patients with HF and/or CLD, and thereby, reduce the number of hospital admissions. We also postulate that home telemonitoring may improve the quality of life of these patients in a way that is cost-effective and acceptable to both, patients and health care professionals.

The **main objective of the study **is to evaluate the impact of a primary care-based telemonitoring intervention on the use of health care resources, specifically on the frequency of hospital admissions.

The **secondary objectives **are:

1. To analyse the impact of the intervention on mortality, duration of hospital stay, use of emergency services, visits to primary care physicians and to specialists, home visits, and telephone calls.

2. To evaluate the telemonitoring procedure in economic terms compared to usual care through a cost-effectiveness analysis. The impact on the quality of life of the participating patients will also be assessed.

3. To assess the degree of satisfaction of the patients/caregivers and health care professionals with the telemonitoring intervention.

## Methods/design

### Main characteristics of the study

#### Study design

**Figure 1:**
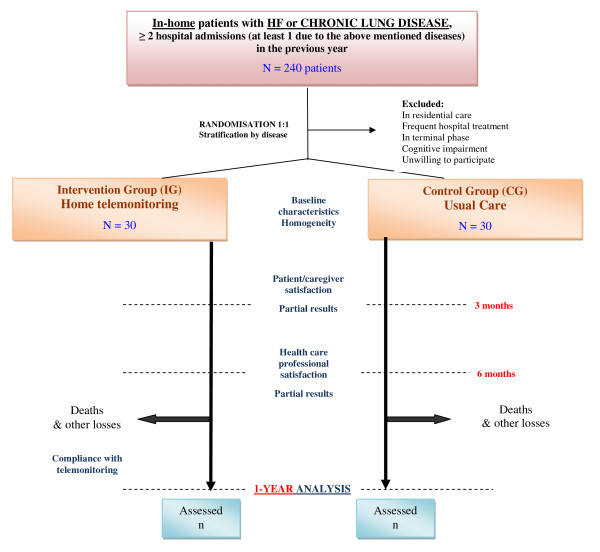
**General design of the randomised controlled trial**.

This is a randomised controlled trial with a one-year follow-up. In the intervention group (IG), in addition to the standard practice, patients will be monitored using a telemonitoring procedure, while the control group (CG) will receive the usual care. Participants will gradually join the study over a period of six months. The recruitment period could be extended to reach the targeted number of patients. Interim data analysis will be carried out after 3 and 6 months of follow-up and the final results will be obtained at 12 months post-randomisation. Figure 1 outlines the general study design.

#### Study area

This study is being carried out across 23 health centres of the Bilbao Primary Care Health Region (Basque Country, Spain). The Health Region serves a catchment population of 390,000 people, of whom around 27% are over 60 years old. The team of professionals in charge of the first level of health care is composed of 218 GPs, 269 nurses and 44 paediatricians. At the hospital level, there are two public referral centres: Basurto Hospital, the main hospital in the area, and Santa Marina Hospital, which is primarily focused on chronic diseases and palliative care.

### Intervention

#### Description of the intervention

In the IG, telemonitoring will consist of daily transfers of the following self-measured clinical parameters: respiratory-rate, heart-rate, blood pressure, blood oxygen saturation using pulse oxymetry, weight and body temperature. Together with the clinical data, patients will also complete a brief questionnaire aimed at assessing the patient's perception of his/her medical and functional condition. The questionnaire also contains items concerning compliance with medication and diet. Health professionals will check the data daily and will routinely contact the patient by phone every two weeks (nursing staff). Other telephone contacts will be carried out, if necessary, if the data received at the primary health care centre are out of the established limits. The telemonitoring system comprises personalised alerts set for each patient, with messages being sent to a Web-based platform when the recorded parameters fall outside established limits (which may be adjusted over time).

We have established standard initial threshold values for all the telemonitored parameters, which can be further modified by health care professionals according to the specific characteristics of the patients. The established threshold values are essential for the monitoring of the patient's condition and for the detection of unusual changes. When the measurements fall outside the established limits, alerts will be triggered via the PDA terminal and the clinical staff will act according to the medical condition of the patient. Alternatively, the patients will be advised to call the emergency services at the weekends and at times when the health centres are closed.

The standard threshold values for alerts are shown in Table 1.

**Table 1:** Established thresholds for the telemonitored parameters.

Telemonitored parameters	Established threshold values
Respiratory-rate	< 12 or >24 breaths per minute (bpm)
Heart-rate	< 50 or >100 beats per minute (bpm)
Systolic blood pressure	< 100 or >160 millimeters of mercury (mmHg)
Diastolic blood pressure,	< 60 or >95 millimeters of mercury (mmHg)
Oxygen saturation	< 95% (this is a frequently adjusted limit for patients with CLD)
Temperature	> 37°C
Weight	Should be notified if there is an increase of 1 Kg in three days
Qualitative questionnaire	Negative answers to the questionnaire which correspond to: feeling worse, breathing difficulties, increased nocturia, oedema, worsening of cough, increased sputum production and a change in sputum colour

In the CG, patients will receive usual care, consisting of regular medical examinations in line with the established programmes for monitoring home-based patients. The frequency of the medical examinations will vary depending on the clinical, social and family situation of each patient. Additionally, the GP and/or nurse will see or call the patient on demand in the event of a deterioration in the medical condition. Note that the IG will receive usual care, as well as the extra telemonitoring specified under the study protocol.

**Figure 2:**
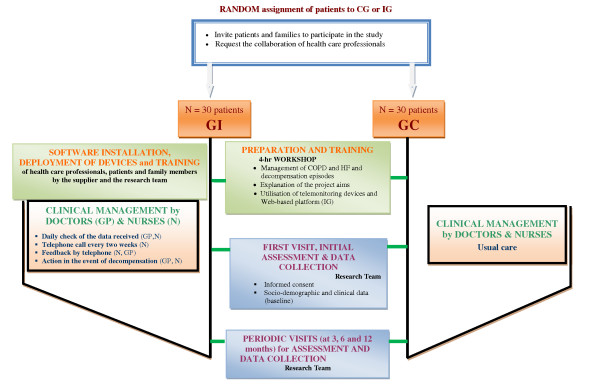
**Key activities for the intervention (IG) and control (CG) groups and personnel involved in the study**.

Prior to the intervention, all GPs and nurses in charge of the care of the participating patients (in both IG and CG) will attend a 4-hour workshop on the management of HF and COPD and early detection of relapses. Additionally, the health care professionals involved with the IG will receive further training on the use of the telemonitoring system. The key activities for the CG and IG are outlined in Figure 2.

#### Technical features of the system and devices for data transfer and reception

**Figure 3:**
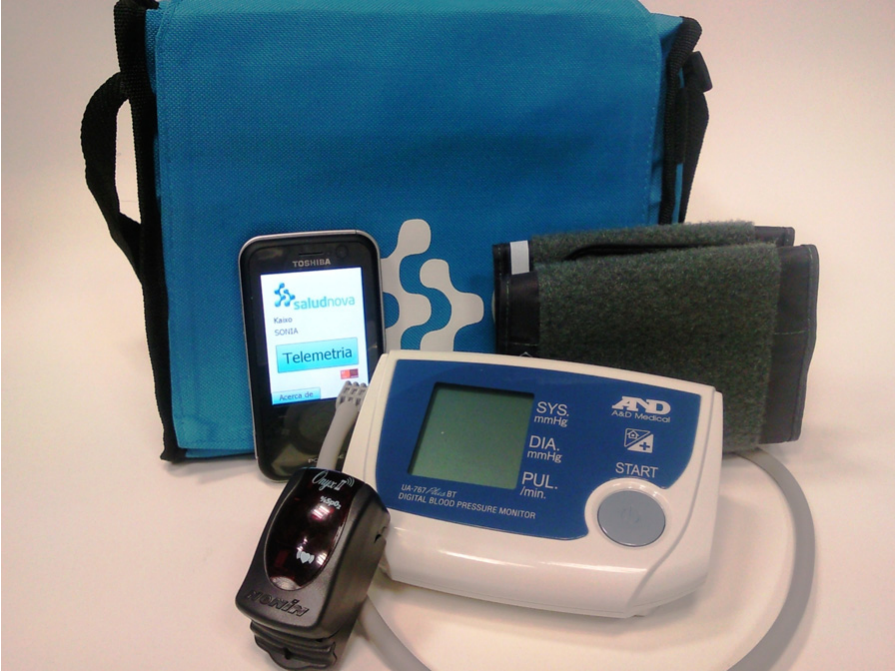
**Telemonitoring kit used by patients in the intervention group**.

**Figure 4:**
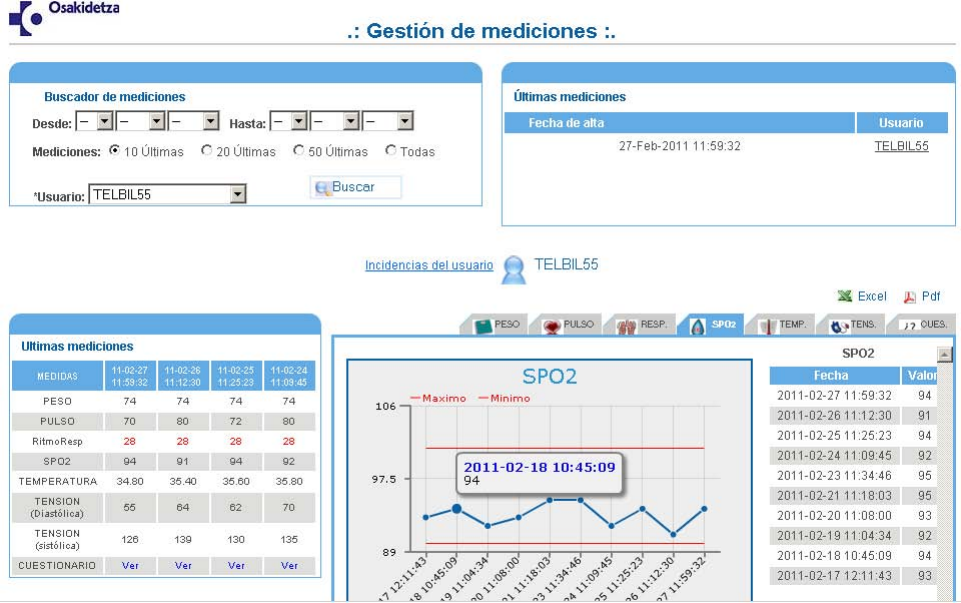
**Web-platform accessed by health care professionals**.

The telemonitoring equipment, supplied by Saludnova [20], consists of a touch screen PDA which runs the Microsoft's Windows Mobile operating system, with wireless Bluetooth sensors to measure blood pressure, heart-rate and oxygen saturation, and manual input of body temperature, respiratory-rate, weight, questionnaires on the medical condition, any perceived changes and a record of compliance with medication and diet. The telemonitoring kit used by patients in the intervention group is shown in Figure 3. The data will be transferred via GPRS to the Web-based platform (Web data manager). The centralised Web management system allows different levels of access depending on the specific roles of the participating professionals (project leaders, GPs/nurses, hospital). The screen of the Web-platform accessed by health care professionals to check the transmitted data is shown in Figure 4. Although the platform can be accessed from any computer connected to the internet, it should be noted that there are terminals available in the consultation rooms of the health centres and in one of the referral hospitals (Santa Marina Hospital), which given its characteristics, often provides care to this type of patients.

### Study Population

#### Eligible patients

The patients included in the study are all attached to the health centres of the Bilbao Primary Health Care Region, are under home care (*i.e*.: in-home patients that receive routine health care at home due to lack of or severe difficulties with mobility), aged 14 or above, have been diagnosed with HF and/or CLD, with two or more hospital admissions during the previous year and at least one of these admissions having been associated with one of the two conditions under study.

#### Exclusion criteria

The following patients are excluded from the study: those in residential care under the supervision of specific health care professionals, those receiving regular monitoring or treatment by specialists or hospital services (such as, rehabilitation programmes, haemodialysis, care in hospital day-care centres, etc.), those in the terminal phases (with a life expectancy under 6 months) of other illnesses (not HF or CLD), patients with cognitive impairment and those not willing to participate in the study.

#### Patient selection process

The lists of patients with two or more hospital admissions for any cause between the 1^st ^of December 2008 and the 1^st ^of December 2009 (12,293 people) was obtained from the hospital records of the two referral hospitals of the Health Region. Subsequently, 240 in-home patients suffering from the clinical conditions under investigation (*i.e*.: HF and/or CLD) were identified using the primary care medical record database ("Osabide"). Fifty nine of these home care patients met all the inclusion criteria and constituted the group of potentially eligible participants for the first phase of the recruitment process. One of the limiting inclusion criterion applied was the fact that at least one of the hospital admissions had to be due to HF or CLD. Progressively, inclusion of additional patients will also be sought through meetings held in the health centres, until the pre-established number of patients is reached.

#### Randomisation

We used a stratified random sampling by disease (HF, CLD or both), which consisted of the random selection of patients for allocation to the control and intervention groups from three containers holding the codes of the initial patients. Subsequent draws were made to maintain the 1:1 ratio between the two study groups and between the three different strata considered.

#### Recruitment of patients

After GPs and nurses from the 23 health centres agreed to join the study, eligible patients were provided with information about the study and were invited to participate and sign a written informed consent form. Additionally, the patients from the IG were given a "Patient information sheet" with detailed information concerning the telemonitoring intervention.

### Study variables

#### Initial assessment and baseline data

The following *socio-demographic data *will be collected for every patient taking part in the study: health centre, assigned GP and nurse, date of birth and age, gender, living arrangements, main caregiver and relationship with the caregiver, level of education and the existence of any adverse social factors (*i.e*.: poverty, social exclusion, loneliness, lack of support, isolation, recent widowhood).

The following *clinical variables *will also be recorded: main disease (HF and/or CLD), degree of severity of the disease (based on the FEV1 - forced expiratory volume in one second- for COPD and on the NYHA - New York Heart Association- classification and the ejection fraction for HF. Severity of illness measures will be collected at the moment of enrolment from the clinical records depending on availability and will be used to ensure comparability between the two groups), requirement for home oxygen therapy, name and list of the drugs taken regularly, adherence to the prescribed medication (compliance) using the four-item self reported scale proposed by Morinsky *et al. *[21], the Barthel Index (BI) [22], the EuroQol questionnaire (EQ-5D) [23-25], and the Zarit Burden Interview (ZBI) [26-28].

#### Outcome measures

All the outcome measures will be collected after 3, 6 and 12 months of follow-up, except for satisfaction with the telemonitoring technology, which will be measured only once (approximately after 3 months from the start of the study). Table 2 shows a summary of the outcome variables that will be evaluated in the present study.

**Table 2:** Summary of the outcome measures analysed in the study.

	Primary outcome measure	Secondary outcome measures
**Mortality**		- Mortality due to any cause
		- Mortality due to HF or CLD
		
**Use of health care resources**	- Hospital admissions due to any cause	**- **Hospital admissions due to HF or CLD
		- Duration of hospital stay
		- Emergency department visits
		- Home visits by GP or nurses
		- Visits to primary care centres by family or caregivers
		- Telephone contacts with professionals
		- Visits to specialist doctors
		
**Clinical efficacy**		- Worsening of the patients' pulmonary and/or heart condition (number of episodes)
		
**Functional status and quality of life**		- Patient Quality of Life (EQ-5D questionnaire)
		- Functional status (Barthel Index)
		- Caregiver burden (Zarit Burden Interview)
		
**Cost-effectiveness**		- Cost-effectiveness (using QALYs)
		
**Satisfaction with the new technology**		- Degree of satisfaction of patients and health care professionals
		
**Compliance**		- Patient and provider compliance with the telemonitoring

##### 1. Primary outcome measure

*Number of hospital admissions due to any cause that occurred in a period of 12 months post-randomisation*.

The hospitalizations will be classified as due to HF, due to CLD (*i.e*., COPD, asthma and other respiratory conditions) and non-cardiorespiratory causes.

##### 2. Secondary outcome measures

2.1 *Duration of hospital stay: *number of bed-days for emergency admissions with a primary diagnosis of HF, CLD (*i.e*., COPD, asthma and other respiratory conditions) and other causes during 12 months after randomisation.

2.2 *Number of hospital admissions due to exacerbation of HF and CLD *(*i.e*., *COPD, asthma and other respiratory conditions*) that occurred in a period of 12 months post-randomisation.

2.3 *Mortality rate: *number of all-cause deaths at 12 months. Cause of death will be taken from the primary and/or secondary care clinical records.

2.4 *Level of use of health resources measured during a period of 12 months after randomisation:*

- Number of emergency department visits for a cardiac or respiratory cause and for all causes.

- Number of home visits (by their GP or nurse).

- Number of primary care visits.

- Number of telephone contacts with primary health care professionals.

- Number of visits to the specialist doctors.

2.5 *Cost-effectiveness analysis*. The costs associated with the health care resources used will be estimated based on the following variables: duration of hospital stay, use of emergency services, purchase and maintenance of telemonitoring devices, number of consultations and time of the health care personnel, as well as the time dedicated by the health care professionals to using the telemonitoring equipment. The effectiveness will be expressed in Quality-Adjusted Life Years (QALY) and will be calculated from the data of the Health Related Quality of Life (HRQL) obtained from the generic EQ-5D questionnaire.

2.6 *Health related quality of life (HRQL)*. The validated Spanish version of the EQ-5D questionnaire will be employed for the quality of life assessment [23-25]. This questionnaire describes the health status in five dimensions (mobility, self-care, usual activities, pain/discomfort, and anxiety/depression) with three possible responses for each item and using the visual analogue scale (VAS) ranging between 0 and 100.

2.7 *Other variables of clinical efficacy: number of episodes of worsening of the pulmonary and/or heart condition*. An episode of worsening or exacerbation is defined as a sustained worsening of the patient's symptoms from a usual stable state that is beyond normal day-to-day variations and is acute in onset. Commonly reported symptoms are oedema, breathlessness and nocturia for HF, and worsening breathlessness, cough, increased sputum production and change in sputum colour for CLD. An episode of worsening is considered a change in these symptoms that requires intervention or control by the physician.

2.8 *Functional status*. The Barthel Index (BI) [22] will be used to measure Activities of Daily Living (ADL). The score on the BI ranges between 0 and 100, with results indicating: total dependence (0-20), severe dependence (21-60), moderate dependence (61-90), little dependence (91-99), and total independence (100).

2.9 *Caregiver burden*. The burden of the family/caregiver will be measured using a validated Spanish version of the Zarit Burden Interview (ZBI) [26-28]. The score ranges from 0 (no burden) to 88 (highest level of burden).

2.10 *Degree of acceptance and satisfaction of patients and health professionals*. The degree of satisfaction of the patients with the telemonitoring intervention will be assessed using a questionnaire based on validated published surveys adapted for this study [29-31]. It consists of 18 items grouped in eight dimensions. The patient satisfaction questionnaires will be administered at the patient's home by an independent female sociologist not involved in the care process. The degree of satisfaction of GPs and nurses participating in the intervention group will be assessed through a qualitative study based on focus group methodology and by means of a specific questionnaire designed *ad hoc*. A Web-based version of the questionnaire for health care professionals will be delivered electronically.

2.11 *Evaluation of the technical performance and compliance with the telemonitoring system*.

Compliance with telemonitoring will be evaluated through the analysis of the frequency of data transmitted by patients and the number of times that health care professionals access the telemonitoring Web-platform. Additionally, the reliability and performance of the telemonitoring system will be assessed (malfunctions of the system, problems concerning the transfer, reception and visualisation of data). The security of the system will also be evaluated (external attacks to the transmission system or server, etc.).

The reasons for the *losses *occurred during the study will be recorded. It is envisaged that such losses may include patients moving house or dying, failure to correctly manage the system, technical problems, requirements for specific health care (for example, patients in need of palliative care at home) and institutionalisation. Additionally, data concerning eligible patients or professionals who declined to participate in the study and the reasons for not participating in the study will be recorded.

### Sample size

Thirty patients will be recruited in each of the study groups, the main constraints being the number of available devices and the number of potential patients fulfilling the inclusion criteria in the Bilbao Primary Care Health Region. Thus, if 30 patients are included in each group, and a 10% loss to follow-up is assumed at 12 months (based on previous analysis of mortality and other causes of loss in patients with similar characteristics), we have estimated a statistical power of 72% to detect significant differences between the CG and the IG in the mean number of total admissions with a level of significance of 5%. For the power calculation, it has been assumed that the mean number of admissions in the CG is 3.5, the standard deviation 1.7, and there will be a 35% decrease of hospitalisations in the IG with respect to the CG [32-34]. The power calculations have been performed with the sample size calculation software Ene 2.0.

### Statistical Analysis

The download, processing and statistical analysis of the data collected during the study will be performed using PASW Statistics 18 software (SPSS). The researchers in charge of the statistical analysis will be blinded to the assignment of the patients to the IG or CG.

#### Description of the baseline characteristics of the patients

The unit of the study is the patient. The following statistics will be used for the descriptive analysis: mean, standard deviation, median and rank for the quantitative variables, and frequency and percentage for the qualitative variables. Socio-demographic and baseline clinical data will be compared between the two groups in order to test the homogeneity. Losses occurring during the study will be analysed and the main baseline characteristics of dropouts will be compared to those who have completed the study. For the comparison of quantitative variables, the Student's t-test and the non-parametric Mann Whitney U test will be used for normally and non-normally distributed data, respectively. The Chi-square or the Fisher's exact tests will be used for the comparison of qualitative variables. The level of statistical significance will be set at p < 0.05.

#### Analysis of the main outcome measure and impact on the use of the health care services

Firstly, at 3, 6 and 12 months follow-up, a descriptive analysis of the primary and secondary outcome measures will be carried out in the two study groups (number of hospital admissions, duration of the hospital stay and mortality rate). The variables related to the use of health care services, clinical efficacy, functional status of the patients (BI), the health related quality of life -HRQL- (EQ-5D) and the family/caregiver burden (ZBI) will also be analysed. Additionally, given that certain variables will have different follow-up periods, the incidence-rates of certain variables, such as mortality, hospital admissions, visits and episodes of worsening will be calculated.

Subsequently, both primary and secondary outcome measures will be compared between the two groups of patients at each follow-up point to test whether significant differences exist. Any changes in the functional condition (BI) or HRQL of the patients (EQ-5D) and caregiver burden (ZBI) with respect to the baseline levels will also be assessed at 3, 6 and 12 months (*i.e*., difference between the follow-up and initial scores). Quantitative variables will be compared using the Student t-test or the non-parametric Mann Whitney U test, while qualitative variables will be assessed using the Chi-Square or Fisher's exact tests. The Student's paired t-test will be used to compare the differences between the baseline scores on the BI, the EQ-5D and the ZBI and the scores obtained at 3, 6 and 12 months follow-up, for each group of patients.

If statistically significant differences are detected between the CG and the IG for the baseline characteristics analysed, the comparisons of the main outcome measures will be adjusted by the corresponding baseline variables. A logistic regression model will be used to compare dichotomous categorical variables (such as, mortality), while a general linear model will be used for quantitative variables (such as, the duration of hospital stay). In the latter case, the dependant variable will be appropriately transformed if it does not fit the normal distribution. Finally, multilevel analysis will be performed to test the effect of the individual primary care professionals involved and the clinical practice site on the results.

Whenever possible, the number needed to treat (NNT) will be calculated for the primary outcome variable (number of hospital admissions).

#### Economic analysis of the telemonitoring intervention

A cost-effectiveness analysis will be undertaken from the perspective of the health care provider. The time horizon will be of one year.

##### Assessment of the effectiveness

The QALYs, calculated from the EQ-5D questionnaire results, will be used as a measure of effectiveness. The QALYs will be estimated by calculating the area under the curve (AUC), which is obtained by summing up the areas of the geometrical shapes obtained by linear interpolation between the utility scores during the study period [35]. In addition, the baseline utility will be controlled using a regression-based adjustment, since there tends to be a strong correlation between the baseline utility of patients and the QALY scores [35].

##### Cost assessment

Only direct unit costs per patient will be calculated. Indirect (caregivers, etc.) and intangible costs will not be considered for the analysis. The specific costs that will be taken into account include: costs directly associated with the telemonitoring intervention (cost of purchase and maintenance of the system, training of professionals in the use of the system, salaries of the health care professionals), costs associated with the home care (visits and telephone calls), costs related to the impact of the telemonitoring program on the necessary resources for the provision of patient care (number of visits to a primary care doctor, visit to the emergency department, number of hospital admissions). The costs will be based on 2009 figures and will be expressed in euros.

Health resources use estimates will be measured considering the following variables: number of telephone consultations (both control calls and calls made as a result of alerts) made or received by health care professionals (by the GP or nurse), average duration of telephone consultations, number of home care visits (both control visits and visits due to alerts) performed by health care professionals (by the general practitioner or nurse), average time spent by health care professionals to check the telemonitoring data daily, number of primary care visit, number of emergency department visits, number of visits to the specialist doctors and number of hospital admissions. The time taken to check telemonitoring data daily or undertaken monitoring telephone calls will be estimated by timing a sample of these tasks.

Resource use estimates will be combined with unit costs per patient obtained as follows: the time spent by health care professionals in made and answered telephone consultations and in checking the telemonitoring data daily, will be assessed based on the salaries for nurses and GPs as reflected in the Basque Health Service wage payment tables; primary care visits, emergency department visits and visits to the specialist doctors, will be amounted in accordance with the Osakidetza billing rates for health care services; hospital admissions will be assessed according to Diagnostic Related Groups (DRGs) for each pathologic condition; telemonitoring equipment (software and devices) will be assessed based on their equivalent annual cost, maintenance and training for their management will be valued using the market price provided by the supplier.

The cost of telecommunications and computer equipment will not be considered, since this is an already implemented infrastructure in the Basque Health System, which is used for many other applications and daily tasks in addition to the telemonitoring process. The cost per patient will be minimal and, therefore, ignored in the costs calculation.

As the comparison is restricted to the programs under study, costs common to both need not be considered as they will not affect the choice between the given programs.

The costs will be based on 2009 figures and will be expressed in euros. The discount-rate used to calculate the equivalent annual cost will be 3%.

##### Presentation of the economic analysis

Once the QALY scores and the costs associated with the two care options under study have been estimated, the incremental cost-effectiveness ratio (ICER) will be calculated to determine which approach is most effective in terms of QALYs. To determine the degree of uncertainty, a sensitivity analysis will be carried out using the non-parametric bootstrapping method [36].

The ICER values estimated using bootstrapping re-sampling, together with the resulting distribution, will be used to estimate the 95% confidence intervals and will be plotted on a cost-effectiveness plane, to determine the quadrant in which the procedure under study lies (dominated, dominant, more costly and more effective or less costly and less effective). The cost-effective ICER proportions that lie, under the threshold widely accepted in Spain (30,000€/QALY) will be plotted using acceptance curves.

#### Analysis of the patient satisfaction with the telemonitoring procedure

The validity and reliability of the patient satisfaction questionnaire will be analysed. A panel of experts in Health Technology Assessment will evaluate the face and content validity of the instrument. Cronbach alpha will be calculated to measure the reliability of each scale. An exploratory factor analysis will be conducted to determine the construct validity. Convergent and divergent validity of the items will be assessed by determining the correlation between each item and the factors.

The scales corresponding to each of the domains of the questionnaire will be calculated by summing up the responses obtained for all items on that scale and will be standardised on a 0 to 100 scale. Given the absence of a gold standard, the criterion-related validity will be studied by determining whether the patients reporting lower levels of satisfaction correlate with higher mortality rates, longer hospital stays, higher number of hospital admissions, higher use of health care resources and more episodes of worsening. In addition, the scales of the questionnaire will be compared by socio-demographic and baseline clinical characteristics. The Student t-test or the analysis of variance will be used for normally distributed data, and the Mann Whitney U test or the Kruskal-Wallis test for non-normally distributed data.

#### Analysis of the technical performance and compliance with the telemonitoring system

Data concerning patient and health care professional compliance with telemonitoring, as well as the reliability, performance and security of the telemonitoring procedure over the course of the study will be described.

### Ethics approval

The study was approved by the Ethics Committee for Scientific Research (CEIC, Basurto Hospital, Bizkaia) on the 16^th ^of December 2009. Patients or relatives gave written informed consent prior to participating in the study.

### Study limitations

Due to the interactive nature of the intervention, it is not possible to blind the health care professionals providing the intervention or the participants involved in the study. Despite the aforementioned limitation, most of the key data are objective and will be obtained from medical registers (hospital admissions, use of health care resources, etc.). Methods have been put in place to double-check the veracity of the data obtained. Moreover, the statisticians in charge of the data analysis will be blinded to group assignment.

The number of patients included in the present study is limited by the available telemonitoring devices. Despite recruitment of a higher number of patients would have been desirable, we have estimated a power of 72% to detect significant differences in the main outcome measure between the two study groups.

## Discussion

The results of this study will shed some light on the effects of telemonitoring for the follow-up and management of chronic patients from a primary care setting. The present investigation will provide data on the clinical efficacy, patient's quality of life and health services costs. The level of acceptance, obstacles and facilitating factors for the use of the new telemonitoring technology will also be explored. Improving the medical support provided to elderly chronic patients and their family/caregivers has the potential to decrease health service usage and increase their quality of life through improved worsening symptom identification and a better continuity of care.

Among the main contributions of the present study we could highlight the following:

1. The telemonitoring intervention will be controlled and managed by the primary health care professionals (GPs and nurses) who routinely see the patients in the health centres. This is a realistic approach that integrates a new health care strategy within the primary health care sector. The study involves all the primary health centres in the Bilbao Health Region and, therefore, the application of ICTs to the management of chronic patients may have a significant impact on the internal organisation of an entire Health Region.

2. The subgroup of patients selected for the study suffers from two of the most commonly targeted diseases for home telecare interventions (*i.e*., HF and CLD). In addition, the patients included in the present study show certain added peculiarities: they are home care patients with frequent hospital admissions and with a higher mean age than that targeted by most other published studies. Therefore, the study may contribute to enhance the understanding of alternative modes of health care provision for medically unstable patients with a high degree of physical and functional deterioration.

3. The present study will also evaluate the acceptance of the new technology by health care professionals and patients/caregivers which could be critical for the adoption of ICTs in the health care sector. In particular, we would like to point out the considerable number of health care professionals that will be involved in the study, which could provide substantial data to thoroughly assess provider satisfaction with telemonitoring technology by means of qualitative and quantitative methods.

4. The study could provide evidence to determine the feasibility of the use of ICT applications by elderly patients with limited computer literacy.

## Competing interests

The authors declare that they have no competing interests.

## Authors' contributions

IML, as the principal investigator, has been involved with and coordinates all the phases and activities of the project. EO has contributed to the drafting and critical revision of the manuscript, and has participated in the conception and design of the study. CC and RA have contributed to the design of the study, assessment of patients and review of documents. JA, MIR and JL have been involved in the conception of the study and are in charge of the institutional relationships and the link with the supplier of the telemonitoring devices. AB and IV have been involved in drafting the statistical analysis and will conduct the statistical analysis of the trial results. JCB has contributed to the drafting of the economic analysis and has participated in the design of the study. ER will contribute to the evaluation of the satisfaction of patients and health care professionals with the telemonitoring technology. All authors have read and approved the final version of the manuscript.

## Pre-publication history

The pre-publication history for this paper can be accessed here:

http://www.biomedcentral.com/1472-6963/11/56/prepub
